# The Forty-Second Annual Commencement of the Baltimore College of Dental Surgery

**Published:** 1882-03

**Authors:** 


### EDITORIAL, ETC.

*The Forty-second Annual Commencement of the Baltimore College of Dental Surgery* was held on Thursday, March 9th, 1882, at Ford's Opera House, in the presence of a large assemblage. The stage was occupied by the Faculty and members of the graduating class, a number of the Board of Visitors, and many invited guests, among the latter being prominent dental and medical practitioners from different parts of the country and Baltimore City. The front of the stage was covered with the customary floral tributes for presentation to the graduates.

The music was by the orchestra of the Opera House, consisting of five selections admirably rendered.

After prayer by the Rev. Joseph B. Stitt, the Dean, Prof. F. J. S. Gorgas recited the authority by which the degree of Doctor of Dental Surgery was conferred, and announced the names of the graduates, forty-seven in number.

The following gentlemen then had the degree of Doctor of Dental Surgery conferred upon them by Prof. Gorgas:

| | |
|---|---|
| Henry Seraphim Abendschein, | *Maryland.* |
| Charles Lee Alexander, | *North Carolina.* |
| Pedro A. Arcentales, | *South America.* |
| Julius Alonzo Ballentine, | *North Carolina.* |
| John S. Bizzell, | *North Carolina.* |
| Gordon H. Claude, | *Maryland.* |
| W. Connor Cleckley, | *South Carolina.* |

| | |
|---|---|
| Genaro W. Cooke, | *South America.* |
| Charles William Daly, | *B. West Indies.* |
| Amos Chapin Daniels, | *Pennsylvania.* |
| Willie Ferdinand Davison, | *Virginia.* |
| William Harper De Ford, | *Dist. Columbia.* |
| Charles F. Dinger, | *Maryland.* |
| Louis Picquet Dotterer, | *South Carolina.* |
| Thomas S. Eader, | *Maryland.* |
| Wallace W. Freeman, | *Maryland.* |
| Ferdinand Samuel Gorgas, | *Pennsylvania.* |
| G. Ashman Hamill, | *West Virginia.* |
| Irby Hardy, | *Virginia.* |
| Lewis James Harmanson, | *Virginia.* |
| William Hepburn, Jr., | *New York.* |
| George Wesley Hunt, | *Pennsylvania.* |
| Philip Fletcher Laugenour, | *North Carolina.* |
| Archie McAlpine, | *Pennsylvania.* |
| James P. McDonald, | *Mississippi.* |
| John Miller, | *New York.* |
| George Edward Morrow, | *Maryland.* |
| Steuart Brown Muncaster, | *Dist. Columbia.* |
| Gustavus North, | *Iowa.* |
| George A. Patrick, | *Georgia.* |
| Hugh Pirkey, | *Virginia.* |
| W. Chalmers Ralston, | *Pennsylvania.* |
| B. Taylor Read, | *New York.* |
| Norman J. Roberts, | *Illinois.* |
| Jose Justiano Sanjurjo, | *West Indies.* |
| Samuel P. Sharp, | *Tennessee.* |
| James E. Shields, | *North Carolina.* |
| Charles Alfred Slocum, | *New York.* |
| Henderson Snell, | *North Carolina.* |
| Mordecai Gist Sykes, | *Maryland.* |
| George G. Taylor, | *Virginia.* |
| Lewellen C. Tucker, | *Virginia.* |
| T. John Welch, | *Virginia.* |
| B. H. Whittington, | *Maryland.* |
| Robert Campbell Williams, M. D., | *South Carolina.* |
| John M. Wilson, | *Pennsylvania.* |
| Cincero Reneta Yearick. | *Ohio.* |

After the conferring of the degrees, Dr. M. W. Foster, the President of the Board of Visitors, presented the Collegiate prizes, all of which, except the first prize, had been awarded by Committees of the Board of Visitors appointed for the [illegible]r-pose. The awards were as follows:

1st. *The College Prize*, a very handsome gold medal, was awarded to John S. Bizzell, of North Carolina, for the highest number of votes at the final examination. Louis P. Dotterer, of South Carolina, received honorable mention.

2nd. *A Dental Engine*, the S. S. White prize, was awarded to Jose J. Sanjurjo, of Port Rico, West Indies, for best work in whole mouth, with essay on preparation of mouth and filling teeth. Archie McAlpine, of Pennsylvania, Norman J. Roberts, of Illinois, and Mordecai G. Sykes, of Maryland, received honorable mention.

3rd. *Set of Harris' Forceps*, the Snowden & Cowman prize, was awarded to Jose J. Sanjurjo, of Porto Rico, W. I., for the best upper and lower set in celluloid, with essay on treatment of exposed pulps and dead teeth. John Miller, of New York, received honorable mention.

4th. *Set of Varney's Points*, the S. S. White prize, was awarded to Charles L. Alexander, of North Carolina, for the best piece of plate work, with essay on Mechanical Dentistry.

To Samuel P. Sharp, of Tennessee, was awarded a *set of Jack's Chisels*, the J. Curtiss Smythe prize, for the second best piece of plate work, with essay on Mechanical Dentistry.

The annual oration was then delivered by Prof. Richard F. Gundry, M. D., Superintendent of the Maryland Insane Asylum, and was replete with practical advice, and the importance of dental science to the human race.

The Class Valedictory was delivered by Dr. John S. Bizzell, of North Carolina, in a manner which was commended in the highest terms by very many of those who listened to it. The exercises closed with the benediction by Dr. R. V. W. Clarkson, of Illinois, of the class of 1846. The number of graduates was 47, and the number of matriculants 93.

*Alumni Meeting.*—The Alumni Association met at the college at 10 A. M., Thursday March 9th, Prof. F. J. S. Gorgas, President, Dr. W. S. Harban, of Washington, Secretary, Dr. B. Holly Smith, Jr., Orator. The following officers were elected for the ensuing year: President, Dr. J. B. Hodgkin, of Washington; Vice Presidents, Drs. L. De L. Gorgas and C. C. Harris, o Baltimore; Treasurer, Dr. E. B. Bliss, of Washington; Corresponding Secretary, Dr. Charles Steel, of Richmond; Recording Secretary, Dr. James E. Shreeve, of Ellicott City; Orator, Dr

Charles F. Dinger, of Baltimore. A reunion of the Alumni took place at Wagner's Green House in the evening and was greatly enjoyed by a large company.

